# The institutional repository landscape in medical schools and academic health centers: a 2018 snapshot view and analysis

**DOI:** 10.5195/jmla.2019.653

**Published:** 2019-10-01

**Authors:** Daniel G. Kipnis, Lisa A. Palmer, Ramune K. Kubilius

**Affiliations:** Life Sciences Librarian, Reference and Information Services, Campbell Library, Rowan University, Glassboro, NJ, kipnisd@rowan.edu; Institutional Repository Librarian, Lamar Soutter Library, University of Massachusetts Medical School, Worcester, MA, lisa.palmer@umassmed.edu; Collection Development/Special Projects Librarian, Galter Health Sciences Library & Learning Center, Northwestern University Feinberg School of Medicine, Chicago, IL, r-kubilius@northwestern.edu

## Abstract

**Objective:**

This study uses survey research methods to gain a deeper understanding of the institutional repository (IR) landscape in medical schools and academic health centers.

**Methods:**

Members of the Association of Academic Health Sciences Libraries (AAHSL) were surveyed about their IRs. The authors used a mixed-methods approach of a survey and qualitative content analysis to identify common themes.

**Results:**

Survey results indicate that a large majority of responding medical schools and academic health centers have or are implementing an IR (35 out of 50, 70%). Of these, 60% (21 institutions) participate in an institution-wide IR rather than administer their own repositories. Much of the archived content is grey literature that has not already been published, but the percentage of original content varies greatly among institutions. The majority (57.1%) of respondent institutions are not considering an open access policy or mandate. Most institutions (71.4%) reported that repository staff are depositing materials on behalf of users. DSpace and bepress Digital Commons are the most popular repository platforms in this community. The planned enhancements that were most frequently reported were implementing a discovery layer and ORCID integration. The majority of respondents (54.3%) do not plan to migrate to a different platform in the foreseeable future. Analysis of respondent comments identified the following themes: integration, redundancy, and reporting; alternatives and exploration; uniqueness; participation; and funding and operations.

**Conclusions:**

The study results capture a view of the IR landscape in medical schools and academic health centers and help readers understand what services their peers have in place as well as their plans for future developments.

## INTRODUCTION

Institutional repositories (IRs) have been integrated into the services that medical libraries provide to their user communities for more than a decade. An IR is an online digital archive that organizes, preserves, and provides access to the educational, scholarly, and research output of an institution. IRs serve as tools to promote open access and to collect, showcase, and disseminate scholarly content produced by an institution, including journal articles, posters and presentations, data sets, and student works such as theses and dissertations. According to OpenDOAR, an authoritative directory of open access repositories, as of April 2019, there were more than 4,100 open access repositories around the world, and 378 of these repositories had health and medicine content [1].

An examination of the published literature indicates that while there is a large and growing body of literature about IRs, much of it international in scope, research on IRs in health sciences libraries is limited. This research on health sciences IRs has predominantly taken the form of case reports describing the challenges and opportunities of launching an IR at a particular institution. For example, Krevit and Crays outlined a pilot program to implement an IR at the Houston Academy of Medicine-Texas Medical Center Library and the University of Texas School of Nursing at Houston to develop what was described as a “multi-institutional repository in a research-intense academic medical environment” [2]. Fay et al. described the process for setting up and populating an IR for the large Aurora Health Care system in Wisconsin [3]. Also, Ilik et al. reported on the development and launch of a next-generation repository for the nontraditional scholarly outputs of Northwestern Medicine [4].

Several other studies have employed surveys on IRs in the health sciences environment. Pickett and Knapp surveyed 229 health sciences libraries worldwide about their digital collections and found that approximately half had IRs in 2013 [5]. Loan and Sheikh analyzed 254 health and medical repositories utilizing the information listed in OpenDOAR [6]. In addition, Odell et al. shared findings from a survey that they conducted at their institution that demonstrated that medical faculty were not responsive to changes in scholarly communication, which impacted their self-archiving activities in the IR [7].

The Association of Academic Health Sciences Libraries (AAHSL) began periodically surveying its members about their IRs beginning in 2005 and published some early data and observations [8]. Since then, however, few analyses have been published. Palmer’s article presented a view of the state of medical IRs and trends, including brief statistics that AAHSL compiled in 2010 [9]. This view was later updated with statistics from AAHSL’s 2014 member survey, which found that close to 56% of AAHSL libraries reported offering IR services [10]. The latest member survey with data from fiscal year 2017, indicated that close to 60% of AAHSL libraries planned to continue offering IR services and another 8% planned to add IR services within the next 12 months [11].

The purpose of this study was to gather more detailed information than what is currently available and establish a snapshot view of the IR landscape specific to medical schools and academic health sciences centers. This study establishes baseline information about the roles of, characteristics of, and future plans for IRs in this setting. This is an optimal time for gathering data from academic health sciences libraries regarding the current state of IRs and their projections about the near future. Recent developments such as Elsevier’s acquisition of bepress Digital Commons, a popular IR platform, and the growing number of mandates and calls for sharing and preserving research and scholarly outputs have led to increased interest in IR platform solutions and collaborations among libraries of all types [12, 13]. The scholarly communication environment is changing significantly as commercial publishers create and invest in products that support all stages of the research life cycle. Therefore, this study contributes to the professional literature and is directly relevant to the delivery of scholarly communication services in medical libraries.

## METHODS

The goal of this research was to establish a snapshot view of the IR landscape specific to medical schools and academic health sciences centers. In December 2017 through January 2018, the authors surveyed 153 medical libraries that were AAHSL members. AAHSL members were chosen as the survey group because this is the major membership association for academic medical libraries; its member libraries serve the accredited US and Canadian medical schools belonging to the Association of American Medical Colleges. Each library is typically represented by library directors and associate directors. We felt that this approach would provide more complete and accurate information compared to surveying individual medical librarians about the IRs at their institutions.

This study was exempted from review by the Institutional Review Board of the University of Massachusetts Medical School and was determined not to be human subjects research. A twenty-one-question online survey was developed (supplemental Appendix A), and an invitation to participate was sent through the AAHSL email discussion list. The survey opened on December 7, 2017, and data collection continued through January 8, 2018. Study data were collected and managed using Research Electronic Data Capture (REDCap) [14], hosted by the University of Massachusetts Medical School. Respondents were asked to identify their institutions to ensure that one response per library was recorded, and identifying information was filtered from data that were exported for analysis. Only complete responses were analyzed. Respondents had the opportunity to comment on open-ended questions throughout the survey. Each author qualitatively analyzed these comments independently to identify common themes.

## RESULTS

### Response rates

Of the 153 AAHSL member libraries that were sent an email invitation, 63 responded to the survey, for a response rate of 41%. Other online surveys of AAHSL member libraries have had response rates between 33% and 49% [15–18]. Ten incomplete responses were considered unusable and were excluded. Three libraries responded to the survey twice and were contacted by email for clarification about which response to use. The remaining 50 responses (33% of AAHSL members) were used for analysis.

### Current status of institutional repositories (IRs)

Most (70%) respondents indicated that they currently used or were implementing an IR (Table 1). This finding was in line with AAHSL’s fiscal year 2017 member survey results, which found that 68% are planning to continue offering or add IR services [11].

**Table 1:** Current status of an institutional repository (IR) (n=50)

Status	Frequency	Response rate
Yes, the institutional repository (IR) is live and publicly available	34	68%
Yes, we have selected/licensed/developed an IR platform and are in the process of implementing	1	2%
Not yet, we are in the procurement or evaluation process	7	14%
No, we do not use an IR and are not considering one	8	16%

The 15 responding institutions that did not have IRs were asked to explain further and then exited from the survey. Their explanations included financial considerations, not a high priority, lack of demand from the community, lack of support from administration, and lack of staff. Many of these respondents indicated that discussions were ongoing.

Of the 35 responding institutions with IRs, 60% participated in an institution-wide IR and 40% administered their own health sciences IR. Respondents with institution-wide IRs were asked to describe the relationship of the health sciences library with the IR. Their comments revealed varying levels of responsibility, administrative access, and roles in depositing content. Three institutions noted that the health sciences library started or currently runs the institution-wide IR.

**Figure 1:**
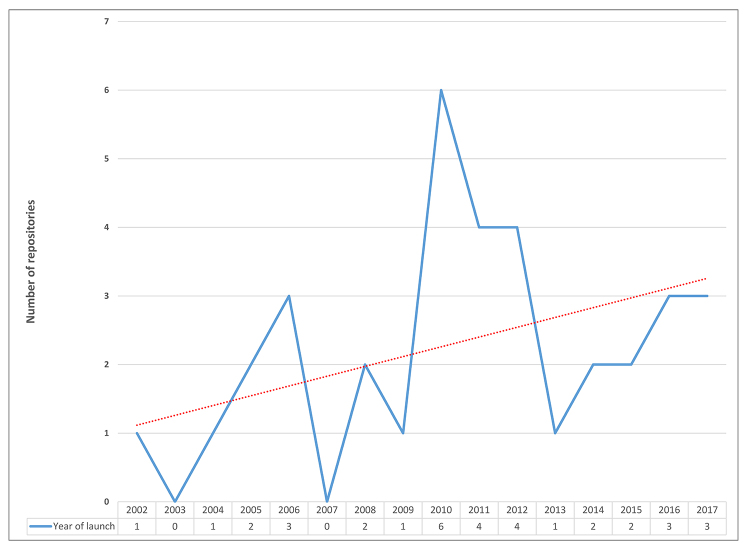
Year of institutional repository (IR) launch (n=35)

The respondents included AAHSL institutions that launched their IRs as early as 2002 and others as late as 2017 (Figure 1). Among this sample, 2010–2012 was the most popular period for beginning an IR, with six repositories launched in 2010, four in 2011, and four in 2012. More recently, three repositories were launched in both 2016 and 2017, continuing the upward trend over the entire time span.

### IR collection size and content

Given the large variation in the age of the IRs in this sample, we expected some disparity in the number of unique digital objects housed in each IR. Respondents were asked to estimate the number of unique digital objects currently in their IRs, including those with embargoes, campus access only, and metadata only. Respondents participating in institution-wide IRs were asked to estimate the number of objects representing health sciences content (e.g., medical, nursing, pharmacy, allied health). The number of unique objects ranged from 50 to 115,246 objects, with a median of 11,738.5 objects.

Respondents were asked about the resource types deposited into their IRs and to select all that applied (Table 2 in supplementary Appendix B). The most popular resource types were dissertations and theses (80%) and journal articles (80%). Smaller percentages of responding institutions indicated that they archived grand rounds presentations (14%), patient education materials (11%), and lab notebooks (3%). Additional types of materials that respondents mentioned in comments included software, literary magazines, student course materials, course catalogues, recordings, music recitals, and patents.

**Figure 2:**
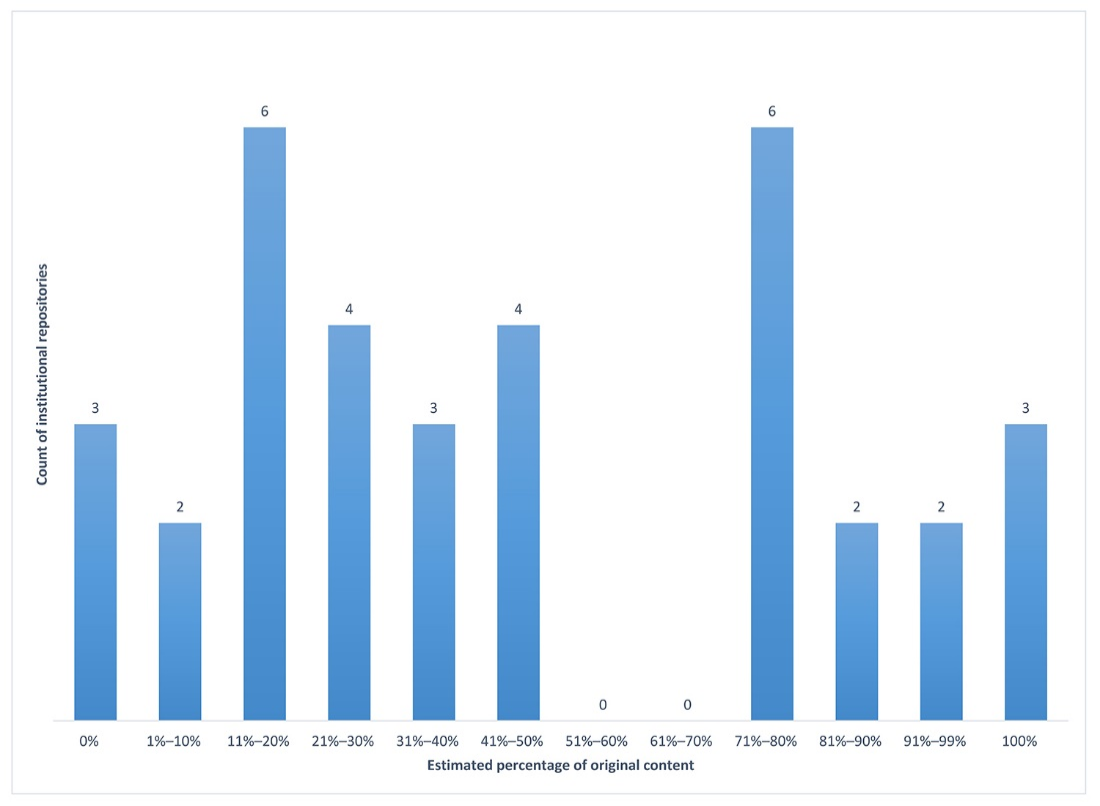
Estimated percentage of original content in IRs (n=35)

Much of the content deposited into these IRs was “grey literature” that had not been published in traditional academic publishing venues (Table 2 in supplementary Appendix B). Respondents were asked to estimate the percentage of content in their IRs that was “original” (i.e., first published in the IR). The survey question listed some examples of original content: open educational resources, journals published through the IR, theses and dissertations, and data sets. Most responding institutions estimated 50% or less original content (Figure 2). Notably, 6 libraries were at the extreme ends of the spectrum: 3 libraries with no original content and 3 libraries with 100% original content. The percentages reported most frequently were 11%–20% and 71%–80%. These results indicated a wide variety of collection development policies for IRs and demonstrated that AAHSL libraries were serving as stewards of institutional grey literature and other scholarly products.

It is relevant to note that the Digital Commons platform from bepress evolved from editorial management software and includes functionality for publishing peer-reviewed journals, which is not widely available in DSpace and other platforms. However, this factor alone did not explain the wide variation in original content estimates. The average percentage of original content reported by institutions using Digital Commons was 50%, compared to 48% for institutions using DSpace. This percentage was lower for both platforms among respondents who reported “peer-reviewed journals” as a resource type: 47% and 36%, respectively.

### IR administration and staffing

Respondents were asked about their methods for populating the IRs with content. When asked to identify the primary deposit method, 71% reported that repository staff deposit materials on behalf of their users. This was consistent with what was reported in the literature about low rates of author self-deposit (“self-archiving”) in IRs, especially in the health sciences [7]. Another 14% used “mediated self-deposit” as the primary method, meaning that authorized users could deposit materials, but repository staff reviewed, approved, and performed final posting for all materials. Only 11% of respondents principally utilized a self-deposit method that was unmediated by repository staff. One library (3%) identified “other deposit method” as the primary method and explained that they harvest content from PubMed and other databases.

Given this high level of staff mediation in the deposit process, how are academic health sciences libraries staffing their IRs? Most responding institutions (66%) reported having a repository manager, although it was clear that many other types of library staff also participated in the workflow (Table 3 in supplementary Appendix B). Additional types of staffing that respondents mentioned in comments included nonlibrary administrators trained to upload and approve content, archival staff, and librarians who provided support for copyright, scholarly communication, and data management.

More than half of the institutions (60%) had 1 or fewer full-time staff working on the IR, with 0 full-time staff reported most frequently (26%). However, 5 IRs (14%) were staffed by 5 or more full-time staff. When asked how many total hours staff collectively spent on repository tasks in a typical week, the ranges that were reported most frequently were 6–10 hours (20%) and more than 20 hours (20%). Respondents noted in the comments that staffing and time allocated to the IR were related to factors such as the IR platform, the role that the health sciences library plays in administering the IR, staff changes due to turnover, and current projects.

### Open access policies

Respondents were asked about the existence of an open access policy or mandate at their institutions, defined as a “policy or mandate [that] requires researchers to provide open access to their peer-reviewed research articles by depositing them in an open access repository.” The majority of institutions (57%) were not considering a policy or mandate at that time, 26% had a policy or mandate that was live across the institution, and 17% were considering such a policy or mandate.

Respondents with open access policies were asked how the policy impacted the operation or workflow of their IRs. As seen in the following excerpts from comments, some have found the policy to be essential and beneficial, while others have found that the policy has not had a big impact.

Essential to the streamlined workflow of the IR, and allows absolute paper trail of the required permissions and licenses.

The implementation of the [open access] OA policy drastically increased the amount of unique digital objects in the IR.

It is not actively enforced and has not led to an uptick in faculty works being submitted to our IR.

It didn’t really affect things except maybe we put less in because we don’t want to duplicate what is in PMC.

### IR platforms and future plans

**Figure 3:**
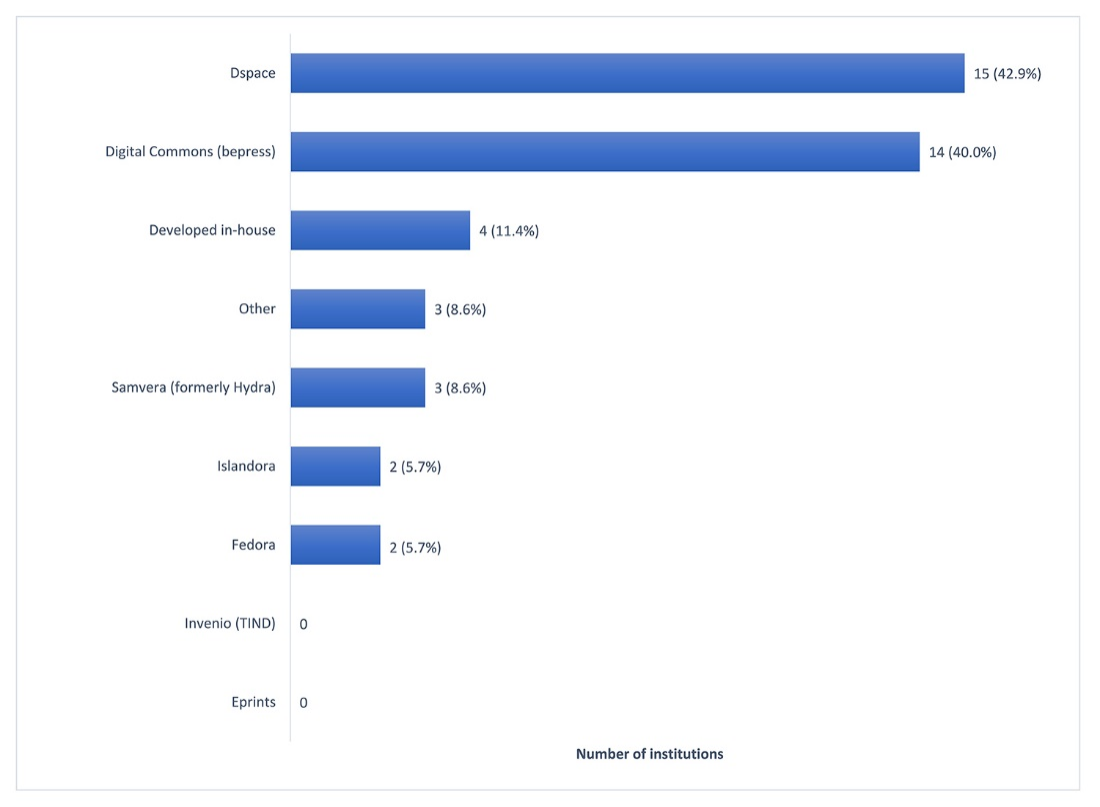
IR platforms currently used (n=35) Respondents selected all options that applied.

Respondents were asked a series of questions about the current platforms for their IRs, recent enhancements, and future plans in this area. DSpace and bepress Digital Commons were the two most popular IR platforms (Figure 3), consistent with Luther’s 2018 CHOICE white paper describing the IR landscape [19]. Most respondents utilized community-developed open source software for their IR platforms. Some institutions reported using more than one product, and respondent comments indicated this was typically format-based, for example, a separate repository for data sets or special collections.

**Figure 4:**
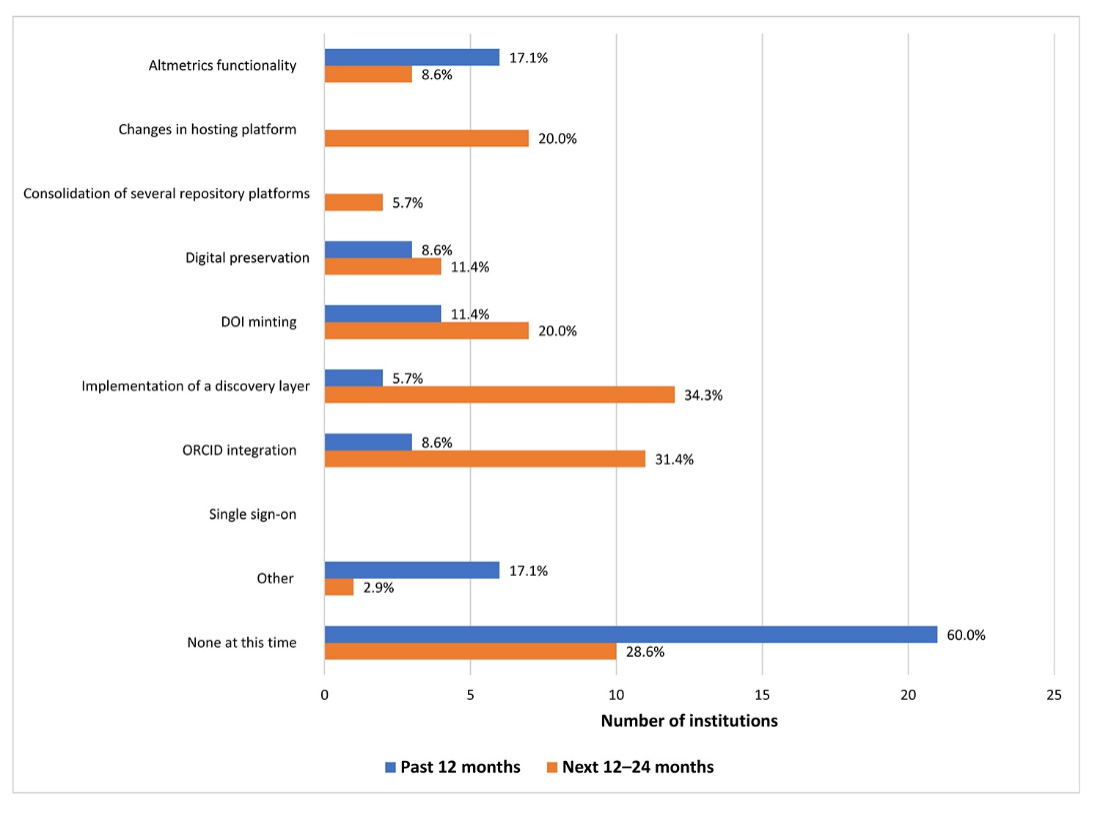
Past and future IR changes and enhancements (n=35) Respondents selected all options that applied.

Respondents were asked about changes to the IR in a number of specific areas and for two time periods: the past twelve months and the next twelve to twenty-four months (Figure 4). Since data collection occurred from December 8, 2017, through January 12, 2018, the two time periods corresponded to 2017 and 2018–2019, respectively. In 2017, the change that was reported most frequently was “none at this time.” For those that implemented changes, the most common change from the list of specific areas was adding altmetrics functionality; as one commenter noted: “We were interested in being able to share any social impact of student, staff and faculty work in order to better capture how and where the work was being shared.” Additional changes mentioned by respondents in comments included implementing Google Analytics, IR LinkOut in PubMed, SWORD through ProQuest, and a repository redesign.

For 2018–2019, the planned changes that were reported most frequently were implementing a discovery layer (34%) and ORCID integration (31%). Less than a quarter of respondents (20%) were considering changing platforms in this time frame. In their comments, respondents provided various reasons for making these changes, including “To keep it functional and viable,” “offer more functionality,” “to better serve our users,” “enhance interoperability,” and “tie in with our faculty productivity system.”

When asked if they anticipated migrating from their current IR platforms in the foreseeable future, most respondents (54%) indicated that they did not plan to migrate to a different platform. Of the other respondents, 6% planned to migrate to a different IR platform within a year, 23% planned to migrate within the next 2–5 years, and 17% were not sure. Thus, it appears that for now, most medical libraries were unlikely to change platforms, although many remained open to considering a migration.

The ten respondents with migrations plans were asked: “to which system will your institution migrate?” Five respondents reported moving away from DSpace (Table 4), with most of the ten appearing to favor open source software for their migration plans.

**Table 4:** Platform migration plans (n=10)

Current platform	Possible migration platforms
Other (Access database) (n=1)	Digital Commons (bepress): 1
Digital Commons (bepress) (n=2)	Islandora: 1, Not sure: 1
DSpace (n=5)	Islandora: 1, Samvera: 1, Not sure: 2, No response: 1
Fedora (n=1)	Invenio: 1
Open repository (n=1)	DSpace: 1

Respondents selected all options that applied.

### Themes in survey respondents’ comments

Finally, respondents were asked to share any other information about their IRs, such as innovative uses, initiatives, concerns, and unique or significant features or collections. In analyzing the responses to this question, as well as comments to earlier questions, we identified five themes: integration, redundancy, and reporting; alternatives and exploration; uniqueness; participation; and funding and operations. Respondents listed concerns that might have been institution-specific but resonated with similarly expressed concerns from other institutions.

### Integration, redundancy, and reporting

There are other efforts by other entities on campus to digitize items into a repository. We need to be aware of those efforts so as not to be redundant.

Also exploring integrations with local faculty profiles system...

Our use of [platform/brand name redacted] which serves as a front-end for our faculty’s research output. Not what I consider an IR however we linked to ORCID and Altmetrics.

We need to develop a good reporting feature and hope to do that in the near future. As of now, we don’t have an easy way of retrieving aggregate count of views/downloads for all items in College of Medicine collections.

### Alternatives and exploration

We are very concerned about the Elsevier acquisition of bepress, but it would be difficult for us to move off the platform given the range of functionality that bepress offers. However, we will be exploring alternatives in 2018. In considering a possible future migration, we have been reviewing our metadata for consistency.

We are in the process of looking into Digital Commons as a replacement for the Access Database. We would like something that allows other users to add publications instead of just the librarians. We would also like more reporting options, and the ability to add the full text from the libraries’ subscriptions.

### Uniqueness

Overall, the School of Medicine is responsible for (roughly) 70% of the total journal articles in the IR and is a major contributor to the university’s OA policy. There are several unique School of Medicine collections within our institutional IR.

Many of our collections are comprised of digitized content from the archives, but we also have and *[sic]* established ETD collection…and are investigating a new nursing presence in the IR for 2018.

Our IR is both a publishing platform for journals and book series, as well as for academic units to host working papers and other types of documents, and for individuals to deposit scholarly “postprints”, or previously published works as we call them...There is a Univerity-wide *[sic]* open access policy under review for theses and dissertations. Assuming it passes, our students’ ETDs will be freely available in the IR.

### Participation

Faculty participation largely depends on open access policy and cultural change.

Many of the top journal titles that our faculty and researchers publish in do not allow for IR deposit (we use the SHERPA/RoMEO database as part of our article deposit workflow). That, combined with the lack of an IR deposit mandate at the institutional level, does limit participation in our IR.

### Funding and operations

Funding to support infrastructure and human resources is always lacking which could dampen the impact of the IR.

We are not considering getting an IR due solely to financial considerations.

Two collections that are managed by health sciences liaisons total 850 items...I would love to be able to provide additional numbers but until I get my vacant position hired, I am not able to spend the time analyzing the spreadsheet metadata generated by the repository reports to figure out what material is health sciences-related and what isn’t.

The IR is a small part of my job, perhaps 5%.

Cataloging librarian is doing the IR work for us.

IR manager splits time between IR, [interlibrary loan] ILL, data catalog and other projects. Metadata librarians too allocate their time to work on IR. Other techs and library interns are a major source for submission and digitization.

Main library hosts IR, they have one dedicated staff person with 2 student workers who do IR work.

## DISCUSSION

The results of this study provide valuable information about the availability of, characteristics of, and future plans for IRs in medical schools and academic health centers. This study addresses a gap in the professional literature by gathering granular and up-to-date information from AAHSL member libraries about their IRs, allowing us to look at trends and acquire a generalized view beyond individual cases and pilot studies [2–4], website and directory surveys [5, 6], and brief availability statistics that have been compiled by AAHSL [11].

Our finding that 70% of the 50 responding AAHSL member libraries indicated that they currently used or were implementing an IR aligned with results of the most recent official AAHSL survey in 2017, which found that 68% of member libraries were planning to continue offering or add IR services [11]. Institutions that did not currently have IRs commented that there were numerous barriers such as financial considerations and lack of community demand, administration support, and staffing.

In this study, we looked for patterns through the prism of health sciences IRs. Over half (60%) of respondents participated in a shared IR with other libraries at their institutions. About a quarter (26%) of respondents were from an institution that adopted an open access mandate or policy, although there was not a clear consensus on the impact of such policies on an IR. The survey results also demonstrated that resources archived in medical IRs were diverse, from prominent content such as dissertations, theses, and journal articles to less common content such as lab notebooks. Thus, IRs were providing a medium and an opportunity for medical schools and academic health centers to collect, curate, and archive grey literature. Respondents were focusing their efforts on unique repository features and collections that added value or showcased health sciences content. Health sciences institutions might be similar to participants of the 2017 Coalition for Networked Information (CNI) Executive Roundtable on “Rethinking Institutional Repository Strategies,” who saw IRs as a “spectrum of services” that made up stewardship strategy [13].

IRs in academic medical libraries appeared to have much in commons with IRs in other academic environments, when compared to the findings of Luther’s recent study of mostly academic institutions in North America [19]. We found that DSpace and bepress Digital Commons were the most popular software platforms used by AAHSL institutions (43% and 40%, respectively). This result was comparable to Luther’s finding that bepress Digital Commons, CONTENTdm, and DSpace were the top platforms (58%, 27%, and 26%, respectively). Most IRs (60%) were managed by 1 or fewer full-time employees, which was also the case for a large percentage of Luther’s respondents (45%).

Medical librarians expressed concerns about ensuring that their IR initiatives harmonized with institutional infrastructure and reporting initiatives, and were not redundant. Recruiting community content for their IRs appeared to be problematic for many libraries. Sustaining financial and operational support over time was also a challenge. Most respondents had an IR manager, but many expressed concerns about insufficient staffing levels for their IRs. These concerns about the role and sustainability of IRs aligned with Luther’s data and analysis of the scholarly communication ecosystem and the evolving role of libraries and repositories [19].

Institutions are watching and responding to new developments in the industry as evidenced in the list of reported enhancements and plans to migrate to a different or next generation IR. The data revealed some interesting views and plans on platform migration. According to the 2017 CNI Executive Roundtable, there were 3 strategies that institutions were taking to move their repositories to the next stage: consolidate them into fewer platforms, migrate to a platform new to the institution, and implement a cross-platform discovery tool [13]. We found that 29% of institutions with IRs reported plans to migrate to a new platform in the next 1–5 years, similar to Luther’s finding that 24% of respondents indicated they planned to migrate in the next 1–3 years [19]. Additionally, we found that 34% of institutions with IRs planned to implement a discovery tool in the next 12–24 months and that 31% planned an ORCID integration project.

Because this study surveyed 153 US and Canadian libraries that were AAHSL members in December 2017, this was a small sample compared to the IR community at large and globally. Although we received a healthy response rate for the targeted audience, our results may not necessarily be generalizable to all medical libraries.

An area for further work is to target a much larger and more diverse survey population, such as the Medical Library Association (MLA), which includes both individual and institutional members of medical and other academic health schools, hospitals, corporations, and research centers. This could generate a larger sample size and would provide more diverse perspectives regarding the status of medical IRs. Also, more granularity and perspective could be achieved if the methodology included other research modalities such as interviews.

## CONCLUSION

Worldwide, the number of IRs is growing, and this same upward trend can be noted for IRs in academic health sciences centers. With the emergence of the second-generation IR movement led by the Coalition of Open Access Repositories (COAR), many medical and academic health institutions continue to develop their IRs [20]. They are also looking to see what is on the horizon, during a time of substantial transition and consolidation, with regard to partnerships, buyouts, and the evolution of scholarly communication services and tools. In this evolving IR landscape, it is important to see what other medical libraries are doing to inform the development of our own services and programs. The present study provides detailed information from AAHSL member libraries about the roles, characteristics, sustainability challenges, future plans, and common concerns for IRs in the academic health sciences community. Our results help institutions understand what services their peers have in place and their plans for the evolution of their medical IRs.

## DATA AVAILABILITY STATEMENT

The de-identified dataset and supporting files are available in the University of Massachusetts Medical School’s IR, eScholarship@UMMS, at https://dx.doi.org/10.13028/4t76-4325.

## SUPPLEMENTAL FILES

Appendix A Survey instrument

Appendix B Tables 2 and 3

## 

**Daniel G. Kipnis, MSI**, kipnisd@rowan.edu, https://orcid.org/0000-0002-4589-5106, Life Sciences Librarian, Reference and Information Services, Campbell Library, Rowan University, Glassboro, NJ

**Lisa A. Palmer, MSLS, AHIP**, lisa.palmer@umassmed.edu, https://orcid.org/0000-0002-4116-1279, Institutional Repository Librarian, Lamar Soutter Library, University of Massachusetts Medical School, Worcester, MA

**Ramune K. Kubilius, MALS, AHIP**, r-kubilius@northwestern.edu, https://orcid.org/0000-0002-7881-9396, Collection Development/Special Projects Librarian, Galter Health Sciences Library & Learning Center, Northwestern University Feinberg School of Medicine, Chicago, IL
