# Supplementary material for: The institutional repository landscape in medical schools and academic health centers: a 2018 snapshot view and analysis
Source: J Med Libr Assoc. 2019 Oct 1;107(4):488–98. doi: 10.5195/jmla.2019.653 (PMC6774547; doi:10.5195/jmla.2019.653)
Supplement: Appendix A [file jmla-107-488-s001.pdf]

JMLA Journal of the Medical Library Association

Supplemental content to
J Med Libr Assoc. Oct;107(4):dx.doi.org/10.5195/jmla.2019.653
www.jmla.mlanet.org
© Kipnis, Palmer, Kubilius 2019

The institutional repository landscape in medical schools and academic health centers: a 2018 snapshot view and analysis

Daniel G. Kipnis, MSI; Lisa A. Palmer, MSLS, AHIP; Ramune K. Kubilius, MALS, AHIP

APPENDIX A

Survey instrument

Dear Colleague:

You are invited to participate in a survey about institutional repositories in academic health sciences libraries because your library is a member of the Association of Academic Health Sciences Libraries (AAHSL).

The purpose of this survey is to establish a snapshot view of the institutional repository (IR) landscape specific to medical schools and academic health centers, including their affiliated hospitals. An IR is an online digital archive that organizes, preserves, and provides access to the educational, scholarly, and research output of an institution. Through your participation, we hope to gain a deeper understanding of the role of, characteristics of, and future plans for IRs in academic health sciences libraries.

Please complete only one survey per library. The name of your library will be kept confidential and will only be used to ensure that one response per library is recorded. No personal information will be requested. The results of this study may be used for educational purposes and reported in professional presentations and in the professional literature. However, results will be anonymized and reported in the aggregate, and no individual subject or identifying information will be shared.

The survey should take twenty to thirty minutes to complete. Respondents have the option to save their progress and return later. The survey will be open until January 8, 2018 [extended through Friday, January 12, 2018].

Thank you for sharing and for your time in completing the survey. If there are questions or concerns about this survey or the results, contact one of the investigators.

By proceeding with this survey, you consent to participate in this research study.

Q1. Please identify your institution: (required)

[Dropdown list of AAHSL members]

Q1a. You selected “Other” because you did not find your institution on the list. Please enter your institution. [open response]

Q2. Does your institution currently use an IR? (required)

- o Yes, the IR is live and publicly available
- o Yes, we have selected/licensed/developed an IR platform and are in the process of implementing
- o Not yet, we are in the procurement or evaluation process [skips to Q2a]
- o No, we do not use an IR and are not considering one [skips to Q2a]

Q2a. You answered that you do not use an IR or do not have one yet because you are in the procurement or evaluation process. Please explain. Then press the Submit button, and you will be exited from the survey. [open response, then survey exit]

Supplemental content to
J Med Libr Assoc. Oct;107(4):dx.doi.org/10.5195/jmla.2019.653
www.jmla.mlanet.org
© Kipnis, Palmer, Kubilius 2019

Q3. Does your institution's medical school/health sciences library administer its own IR or does it participate in an institution-wide IR? (required)

- Own health sciences IR
- Participate in institution-wide IR [skips to Q3a]
- Other [skips to Q3b]

Q3a. You answered that the medical school/health sciences library participates in an institution-wide IR. Please describe the medical school/health sciences library's relationship with the institutional IR in terms of depositing content, level of control, etc. [open response]

Q3b. You answered "Other" indicating that the medical school/health sciences library does not administer its own IR or participate in an institution-wide IR. Please explain. [open response]

Q4. In what year was your IR launched and made publicly available? Enter year in YYYY format. (required) [open response]

Q5. Currently, how many unique digital objects are in your IR? If your institution is part of an institution-wide IR, please estimate the number of objects representing health sciences content (for example, medical school, nursing, pharmacy, allied health, etc.). Include all objects, including those with embargoes, campus access only, metadata only, etc. Enter a number, e.g., 1000. Do not include a comma. (required) [open response]

Q6. Based on the current number of items, please estimate the percentage of content that is original (i.e., first published in your repository). Examples might include open educational resources, journals published through the IR, theses and dissertations, data sets, etc. Enter a percentage between 0–100%. (required) [open response]

Q7. How are materials deposited into your institutional repository? Select all that apply. (required)

- Unmediated self-deposit (an authorized user can deposit materials without repository staff approval)
- Mediated self-deposit (an authorized user can deposit materials, but repository staff review, approve, and perform final posting for materials)
- Repository staff deposit (repository staff deposit materials on behalf of users)
- Other deposit method [skips to Q7a]

Q7a. You answered "Other deposit method" to describe how materials are deposited into your repository. Please explain. [open response]

Q8. Identify the primary method by which materials are deposited into your institutional repository: (required)

- Unmediated self-deposit (an authorized user can deposit materials without repository staff approval)
- Mediated self-deposit (an authorized user can deposit materials, but repository staff review, approve, and perform final posting for materials)
- Repository staff deposit (repository staff deposit materials on behalf of users)
- Other deposit method

JMLA Journal of the Medical Library Association

SC
Supplemental content to
J Med Libr Assoc. Oct;107(4):dx.doi.org/10.5195/jmla.2019.653
www.jmla.mlanet.org
© Kipnis, Palmer, Kubilius 2019

Q9. What is the status of an open access policy or mandate at your institution? An open access policy or mandate requires researchers to provide open access to their peer-reviewed research articles by depositing them in an open access repository. (required)

- o Live implementation: Policy or mandate is live across the institution [skips to Q9a]
- o In the process of implementing: A decision has been made and awaits a faculty vote [skips to Q9a]
- o Exploring: Considering a policy or mandate [skips to Q9a]
- o Not considering: Not considering a policy or mandate at this time

Q9a. Please tell us more about the status of an open access policy or mandate at your institution. How does it or how may it affect the operation or workflow of your IR? [open response]

Q10. Does your library have at least one staff member whose primary responsibility is the IR? (required)

- o Yes
- o No

Q11. How is your IR staffed? Select all that apply. (required)

- o Repository manager
- o Library liaisons
- o Library assistants
- o Student workers
- o Collections staff
- o Metadata staff
- o Technical staff
- o Library interns
- o Volunteers
- o No staffing
- o Other [skips to Q11a]

Q11a. You answered "Other" to describe how your IR is staffed. Please explain. [open response]

Q12. Indicate the number of full-time and part-time library staff positions (FTE) who work directly with the IR (e.g., technical, repository administration, training, making deposits, adding or editing metadata, etc.). Estimate to the closest 0.5 position. [skips to Q12a and Q12b]

Q12a. Full-time (required)

- o 0
- o 0.5
- o 1.0
- o 1.5
- o 2.0
- o 2.5
- o 3.0
- o 3.5
- o 4.0
- o 4.5
- o 5.0 or more

SC
Supplemental content to
J Med Libr Assoc. Oct;107(4):dx.doi.org/10.5195/jmla.2019.653
www.jmla.mlanet.org
© Kipnis, Palmer, Kubilius 2019

Q12b. Part-time (required)

- 0
- 0.5
- 1.0
- 1.5
- 2.0
- 2.5
- 3.0
- 3.5
- 4.0
- 4.5
- 5.0 or more

Q13. In a typical week, approximately how many total hours does the library staff collectively spend on IR tasks? (required)

- 0 hours [skips to Q13a]
- 1–2 hours [skips to Q13a]
- 3–5 hours [skips to Q13a]
- 6–10 hours [skips to Q13a]
- 11–15 hours [skips to Q13a]
- 16–20 hours [skips to Q13a]
- More than 20 hours [skips to Q13a]

Q13a. Please add any additional information you would like to share about IR staffing at your institution. [open response]

Q14. Which IR platform(s) does your library currently use? Select all that apply. (required)

- Developed in-house
- Digital Commons (bepress)
- DSpace
- Eprints
- Fedora
- Invenio (TIND)
- Islandora
- Samvera (formerly, Hydra)
- Other [skips to Q14a]

Q14a. You answered "Other" for your IR platform. Please describe. [open response]

Q15. If you are using multiple IR platforms (e.g., Digital Commons and Fedora), please describe your current repository environment. [open response]

SC
Supplemental content to
J Med Libr Assoc. Oct;107(4):dx.doi.org/10.5195/jmla.2019.653
www.jmla.mlanet.org
© Kipnis, Palmer, Kubilius 2019

Q16. Which resource types are deposited in your IR? Select all that apply. (required)

- Blog posts
- Book chapters
- Books (full text)
- Conference proceedings
- Data sets
- Dissertations and theses (full-text)
- Grand rounds presentations
- Historical materials
- Journal articles
- Journal article preprints
- Journal article postprints
- Lab notebooks
- Newsletters
- Open educational resources
- Oral histories
- Patient education materials
- Peer-reviewed journals
- Photographs or photo gallery
- Presentation slides/posters
- Student capstones (presentations or papers)
- Technical or other reports
- White papers
- Other [skips to Q16a]

Q16a. You answered that “Other” resource types are deposited in your IR. Please describe. [open response]

Q17. Does your IR serve as the hosting platform for formal library publishing services that are offered at your institution? (required)

- Yes
- No

Q18. In the past twelve months, were there changes in your institution’s IR with regard to the following (select all that apply): (required)

- Altmetrics functionality [skips to Q18a]
- Change in hosting platform [skips to Q18a]
- Digital preservation (e.g., Portico, LOCKSS, CLOCKSS, PubMed Central) [skips to Q18a]
- Digital object identifier (DOI) minting [skips to Q18a]
- Implementation of discovery or linking capabilities that affect the IR (e.g., Primo, Summon, VuFind, Symplectic Elements) [skips to Q18a]
- Consolidation of several different repository platforms at your institution [skips to Q18a]
- ORCID integration [skips to Q18a]
- Single sign-on [skips to Q18a]
- None at this time
- Other [skips to Q18a]

JMLA Journal of the Medical Library Association

Supplemental content to
J Med Libr Assoc. Oct;107(4):dx.doi.org/10.5195/jmla.2019.653
www.jmla.mlanet.org
© Kipnis, Palmer, Kubilius 2019

Q18a. Please share why you made the changes to your IR that you selected. [open response]

Q19. In the next twelve to twenty-four months, are there plans to make changes in your institution's IR with regard to the following (select all that apply): (required)

- Altmetrics functionality [skips to Q19a]
- Change in hosting platform [skips to Q19a]
- Digital preservation (e.g., Portico, LOCKSS, CLOCKSS, PubMed Central) [skips to Q19a]
- DOI minting [skips to Q19a]
- Implementation of discovery or linking capabilities that affect the IR (e.g., Primo, Summon, VuFind, Symplectic Elements) [skips to Q19a]
- Consolidation of several different repository platforms at your institution [skips to Q19a]
- ORCID integration [skips to Q19a]
- Single sign-on [skips to Q19a]
- None at this time
- Other [skips to Q19a]

Q19a. Please share why you plan to make the changes to your IR that you selected. [open response]

Q20. Do you anticipate that your institution will migrate from your current IR platform(s) in the foreseeable future? (required)

- Yes, within a year [skips to Q20a]
- Yes, within the next two to five years [skips to Q20a]
- No, not in the foreseeable future
- Not sure

Q20a. To which IR system will your institution migrate? Select all that apply.

- Developed in-house
- Digital Commons (bepress)
- DSpace
- Eprints
- Fedora
- Invenio
- Islandora
- Samvera (formerly, Hydra)
- Other
- Not sure

Q21. Finally, please share any other information regarding your IR (innovative uses, initiatives, concerns, unique or significant features or collections, etc.). [open response]
