# Supplementary material for: The institutional repository landscape in medical schools and academic health centers: a 2018 snapshot view and analysis
Source: J Med Libr Assoc. 2019 Oct 1;107(4):488–98. doi: 10.5195/jmla.2019.653 (PMC6774547; doi:10.5195/jmla.2019.653)
Supplement: Appendix B [file jmla-107-488-s002.pdf]

JMLA Journal of the Medical Library Association

Supplemental content to
J Med Libr Assoc. Oct;107(4):dx.doi.org/10.5195/jmla.2019.653
www.jmla.mlanet.org
© Kipnis, Palmer, Kubilius 2019

The institutional repository landscape in medical schools and academic health centers: a 2018 snapshot view and analysis

Daniel G. Kipnis, MSI; Lisa A. Palmer, MSLS, AHIP; Ramune K. Kubilius, MALS, AHIP

APPENDIX B

Tables 2 and 3

Table 2 Resource types deposited in institutional repositories (IRs) (n=35)

Resource type	Frequency	Response rate
Blog posts	8	22.9%
Book chapters	22	62.9%
Books (full text)	17	48.6%
Conference proceedings	20	57.1%
Data sets	17	48.6%
Dissertations and theses (full-text)	28	80.0%
Grand rounds presentations	5	14.3%
Historical materials	22	62.9%
Journal articles	28	80.0%
Journal article preprints	23	65.7%
Journal article postprints	24	68.6%
Lab notebooks	1	2.9%
Newsletters	20	57.1%
Open educational resources	15	42.9%
Oral histories	10	28.6%
Patient education materials	4	11.4%
Peer-reviewed journals	19	54.3%
Photographs or photo gallery	22	62.9%
Presentation slides/posters	23	65.7%
Student capstones (presentations or papers)	19	54.3%
Technical or other reports	22	62.9%
White papers	17	48.6%
Other	10	28.6%

Respondents selected all options that applied.
* This table originally appeared in: Kipnis DG, Palmer LA. Medical institutional repositories in a changing scholarly communication landscape. Against Grain. 2018 Sep;30(4):33–4, 36. (Available from: <https://www.against-the-grain.com/2018/10/v304-medical-institutional-repositories-in-a-changing-scholarly-communication-landscape/>. (cited 14 Jun 2019]).

JMLA Journal of the Medical Library Association

SC
Supplemental content to
J Med Libr Assoc. Oct;107(4):dx.doi.org/10.5195/jmla.2019.653
www.jmla.mlanet.org
© Kipnis, Palmer, Kubilius 2019

Table 3 Staffing in medical IRs (n=35)

Staffing type	Frequency	Response rate
Collections staff	5	14.3%
Library assistants	11	31.4%
Library interns	4	11.4%
Library liaisons	11	31.4%
Metadata staff	9	25.7%
No staffing	1	2.9%
Repository manager	23	65.7%
Student workers	9	25.7%
Technical staff	11	31.4%
Volunteers	1	2.9%
Other	6	17.1%

Respondents selected all options that applied.
* This table originally appeared in: Kipnis DG, Palmer LA. Medical institutional repositories in a changing scholarly communication landscape. Against Grain. 2018 Sep;30(4):33–4, 36. (Available from: <https://www.against-the-grain.com/2018/10/v304-medical-institutional-repositories-in-a-changing-scholarly-communication-landscape/>. (cited 14 Jun 2019]).
